# Trends in prevalence of gestational diabetes mellitus in Zhejiang Province, China, 2016–2018

**DOI:** 10.1186/s12986-020-00539-8

**Published:** 2021-01-19

**Authors:** Meng Wang, Ru-Ying Hu, Wei-Wei Gong, Jin Pan, Fang-Rong Fei, Hao Wang, Xiao-Yan Zhou, Jie-Ming Zhong, Min Yu

**Affiliations:** Zhejiang Provincial Center for Disease Control and Prevention, 3399 Binsheng Road, Hangzhou, 310051 China

**Keywords:** Gestational diabetes mellitus, Prevalence, Epidemiology

## Abstract

**Background:**

Limited population-based studies have investigated the secular trend of prevalence of gestational diabetes mellitus (GDM) in mainland China. Therefore, this study aimed to estimate the prevalence of GDM and time trends in Chinese female population.

**Methods:**

Based on Diabetes Surveillance System of Zhejiang Province, 97,063 diagnosed GDM cases aged 20–50 years were identified from January 1, 2016 to December 31, 2018. Annual prevalence, prevalence rate ratios (PRRs) and average annual percentage change with their 95% confidence intervals (CIs) were reported.

**Results:**

The age-standardized overall prevalence of GDM was reported to be 7.30% (95% CI 7.27–7.33%); 9.13% (95% CI 9.07–9.19%) in urban areas and 6.24% (95% CI 6.21–6.27%) in rural areas. Compared with 20–24 years age group, women in advanced age groups (25–50 years) were at higher risk for GDM (PRRs ranged from 1.37 to 8.95 and the 95% CIs did not include the null). Compared with rural areas, the risk for GDM was higher in urban areas (PRR: 1.69, 95% CI 1.67–1.72). The standardized annual prevalence increased from 6.02% in 2016 to 7.94% in 2018, with an average annual increase of 5.48%, and grew more rapidly in rural than urban areas (11.28% vs. 0.00%).

**Conclusions:**

This study suggested a significant increase in the prevalence of GDM among Chinese female population in Zhejiang province during 2016–2018, especially in women characterized by advanced age and rural areas.

## Background

Gestational diabetes mellitus (GDM) is characterized by high blood glucose levels during any time of pregnancy (although most likely after week 24). Based on up-to-date estimates from the International Diabetes Federation (IDF), 20.4 million or 15.8% of live births to women in 2019 had some form of hyperglycaemia in pregnancy, of which, 83.6% were due to GDM [1]. As one of the most common medical complications in pregnancy, GDM can not only affect the short-term maternal and fetal outcomes [2, 3], but significantly increase the risks of long-term adverse health consequences both for mothers and their offspring, such as metabolic syndrome, cardiovascular disease and obesity [4–8]. Due to different screening strategies, diagnostic criteria and population characteristics, the prevalence of GDM is varied across populations worldwide, ranging from < 1 to 28% [9]. In recent decades, a dramatically rising trend of GDM among pregnant women has been observed in the international studies and the subsequent health and economic burden have drawn much public health attentions [10, 11]. Asia has the largest population and Asian ethnicity has been reported to be one of the most important risk factors for GDM [12, 13], while relevant estimates of secular trends are still scant, especially in the developing countries. With the national health insurance database covering 97% of the population, a study in Korea reported that the prevalence of GDM among women reached 5.7–9.5% during the period 2009–2011[14]. For Chinese population, two independent studies performed across mainland China in 2006 and 2015 showed that the prevalence of GDM was 4.3% and 3.7% [15, 16]. However, due to the heterogeneous in study design, participants and diagnostic criteria, it is difficult to understand the time trend of GDM at the national level. In recent decades, a very few regional studies have attempted to examine the secular trends of GDM among Chinese population. Among them, successive surveys in Tianjin city suggested that the prevalence of GDM increased by 3.5 times (from 2.3 to 8.1%) during 1999–2012 in women living in urban districts [17–19]. Another study of 13,738 GDM cases, conducted in Xiamen, which is an economically developed city, reported a high but relatively stable prevalence of GDM (ranging from 15.5 to 19.9%) among pregnant women during 2012–2017 [20]. Nevertheless, considering the limited sample size, as well as study period and regional representation in these studies, the up-to-date estimates of secular trends in GDM among Chinese women were warranted. The present study aimed to evaluate the GDM prevalence and time trends in Chinese female population during 2016–2018 by age, residence area and calendar year.

## Methods

### Data sources

Data used in this study came from the Diabetes Surveillance System of Zhejiang province, which was a population-based diabetes registry system established in 2001 by Zhejiang Provincial Center for Disease control and Prevention (CDC). At the beginning of establishment, the system covered 30 representative surveillance districts and over 16 million residents. As early as 2009, the surveillance system has extended to all 90 districts throughout the province, with approximately 48 million residents. The surveillance procedures and quality control measures have been described elsewhere [21, 22] and are thus only briefly recounted here. All types of diabetes cases (i.e., type 1, type 2, gestational or other diabetes) were diagnosed and reported by certificated health practitioners in local hospitals and health services centers. Once the diabetes cases were diagnosed, the patients’ information regarding demographics, diagnosis and laboratory indicators were registered in the system within a week. To make sure that only the newly-diagnosed cases were recorded, the patients registered in the system were further verified according to the characteristics of identity card number as well as full name, gender, date of birth (year and month) and region code. For GDM, it is worth noting that if the event recurred in the same patient during another pregnancy, then the case was considered as an independent new record. Later, the confirmed and recorded patients would be followed-up once per year by the health practitioners in local health services centers. All the recorded diabetes cases were coded according to the International Classification of Disease 10th revision (ICD-10).

### GDM cases diagnosis

In this study, GDM cases were diagnosed with the one-step method according to “The Diagnostic Criteria for GDM (2014)” published by the Chinese Medical Association [23]. Specifically, between 24 and 28 weeks of gestation, a diagnostic 2-h 75-g oral glucose tolerance test was carried out among pregnant women. Women were classified as having GDM when one of the following plasma glucose values was reached or exceeded: 0 h, 5.1 mmol/L; 1 h, 10.0 mmol/L; or 2 h, 8.5 mmol/L.

### Number of births

Due to the lack of direct data on births in this study, we used the number of children to evaluate the number of births given the small probability of a multiple birth. The number of children in each year was evaluated according to the number of childbearing female population (20–50 years) in a year multiplied by the fertility rate of population, which is the average number of children that would be born to a woman over her lifetime. The data on female population of childbearing age in each year were obtained from the Zhejiang Provincial Statistics Bureau and were calculated with the female resident number estimated at the beginning and end of each year (i.e., midyear population). The age-specific fertility rates data in Zhejiang province were from the sixth population census in China, 2010.

### Statistical analysis

Descriptive statistics were used to describe the baseline characteristics of 2016–2018 GDM cases included in the present study with frequency and proportion. The crude prevalence was calculated as the number of GDM cases divided by the number of births. The prevalence was calculated for each age group (20–24, 25–29, 30–34, 35–39, 40–44, 45–50 years) and calendar year (2016–2018), stratified by residence area (urban and rural). The standardized prevalence was calculated using the direct standardization method according to the sixth population census in Zhejiang, 2010. To explore the effects of diagnosis year, age and residence area on prevalence, Poisson regression models were conducted with reporting the prevalence rate ratio (PRR) and 95% confidence intervals (CIs). Within the model, the calendar year was treated as a dummy variable. To examine the time trends of T2DM, the average annual percentage change in prevalence was calculated in multivariable Poisson regression model. Meanwhile, within the model, the calendar year was treated as a continuous variable and the statistical significance of the regression coefficient was tested. All analyses were performed using SAS statistical package (version 9.2, SAS Institute, Inc., Cary, NC, USA).

## Results

Overall, 97,063 GDM cases aged 20–50 years diagnosed between January 1, 2016 and December 31, 2018 were included in this analysis. The mean age at diagnosis was 31.37 (4.86) years. The detailed baseline characteristics of GDM cases included in the study were shown in Table 1.

**Table 1:** Baseline characteristics of gestational diabetes mellitus cases included in this study, 2016–2018

	Gestational diabetes mellitus
Number of cases	97,063
Mean age at diagnosis (SD), year	31.37 (4.86)
Age groups at diagnosis, years (%)	
20–24	6,137 (6.32)
25–29	32,525 (33.51)
30–34	32,057 (33.03)
35–39	20,627 (21.25)
40–44	5,410 (5.57)
45–50	307 (0.32)
Residence area	
Urban (%)	48,525 (49.99)
Rural (%)	48,538 (50.01)

### Residence area

During 2016 and 2018, the crude overall prevalence of GDM was 10.01% (95% CI 9.95–10.07%). Standardized overall prevalence of GDM during the same period was estimated at 7.30% (95% CI 7.27–7.33%); 9.13% (95% CI 9.07–9.19%) in urban areas and 6.24% (95% CI 6.21–6.27%) in rural areas (Table 2). After adjusting for the covariates in the Poisson regression models, the risk for GDM was 1.69 times (PRR: 1.69, 95% CI 1.67–1.72) higher in urban areas (Table 3).

**Table 2:** Mean prevalence of gestational diabetes mellitus, 2016–2018

Age at diagnosis (years)	Number of cases	Number of births	Prevalence (%)	95% CI (%)
Overall				
20–24	6137	241,055	2.55	2.48–2.61
25–29	32,525	397,700	8.18	8.09–8.26
30–34	32,057	216,381	14.82	14.67–14.97
35–39	20,627	85,276	24.19	23.90–24.48
40–44	5410	22,028	24.56	23.99–25.13
45–50	307	6813	4.51	4.03–5.02
20–50	97,063	969,252	10.01	9.95–10.07
Standardized prevalence^a^	212,266	2,907,756	7.30	7.27–7.33
Urban areas				
20–24	2644	57,385	4.61	4.44–4.78
25–29	16,364	138,443	11.82	11.65–11.99
30–34	16,671	81,240	20.52	20.24–20.80
35–39	10,253	31,082	32.99	32.46–33.51
40–44	2470	9614	25.69	24.82–26.58
45–50	123	3429	3.59	2.99–4.26
20–50	48,525	321,193	15.11	14.98–15.23
Standardized prevalence^a^	87,975	963,579	9.13	9.07–9.19
Rural areas				
20–24	3493	179,652	1.94	1.88–2.01
25–29	16,161	255,185	6.33	6.24–6.43
30–34	15,386	118,536	12.98	12.79–13.17
35–39	10,374	51,245	20.24	19.90–20.59
40–44	2940	13,695	21.47	20.78–22.17
45–50	184	4292	4.29	3.70–4.94
20–50	48,538	622,605	7.80	7.73–7.86
Standardized prevalence^a^	116,552	1,867,818	6.24	6.21–6.27

*CI* confidence interval

^a^Age-standardized to the 6th population census in Zhejiang Province, 2010

**Table 3:** Prevalence rate ratios (PRR) of gestational diabetes mellitus in relation to calendar year and demographic factors

Characteristic	Urban		Rural		All	
	PRR	95% CI	PRR	95% CI	PRR	95% CI
Year						
2016	Ref		Ref		Ref	
2017	0.99	0.97–1.02	1.21	1.18–1.23	1.09	1.08–1.11
2018	0.97	0.95–0.99	1.22	1.20–1.25	1.09	1.07–1.10
Age						
20–24 years	Ref		Ref		Ref	
25–29 years	2.57	2.46–2.67	3.26	3.14–3.38	3.00	2.92–3.08
30–34 years	4.46	4.28–4.64	6.66	6.42–6.91	5.64	5.49–5.80
35–39 years	7.16	6.86–7.48	10.39	10.00–10.80	8.95	8.69–9.20
40–44 years	5.58	5.28–5.89	11.04	10.51–11.60	8.14	7.85–8.44
45–50 years	0.78	0.65–0.93	2.20	1.90–2.55	1.37	1.22–1.54
Residence area						
Urban					1.69	1.67–1.72
Rural					Ref	

*PRR* prevalence rate ratios, *CI* confidence interval, *Ref.* reference

Multivariable Poisson regression model with the calendar year as a dummy variable

### Age groups

The mean prevalence was significantly different across all age groups, among which, ranged from 2.55 (95% CI 2.48–2.61%) to 24.56% (95% CI 23.99–25.13%) in total; 3.59% (95% CI 2.99–4.26%) to 32.99% (95% CI 32.46–33.51%) in urban areas, and 1.94% (95% CI 1.88–2.01%) to 21.47% (95% CI 20.78–22.17%) in rural areas. The highest GDM prevalence was seen in 40–44 years age group, followed by 35–39, 30–34, 25–29, 45–50, and 20–24 years age groups (Table 2). Compared with 20–24 years age group, 25–29, 30–34, 35–39, 40–44 and 45–50 years age groups were at significantly higher risk for GDM, with the PRR were 3.00 (95% CI 2.92–3.08), 5.64 (95% CI 5.49–5.80), 8.95 (95% CI 8.69–9.20), 8.14 (95% CI 7.85–8.44) and 1.37 (95% CI 1.22–1.54), respectively (Table 3).

### Prevalence time trends

Annual prevalence and average annual percentage changes of prevalence were shown in Tables 4 and 5. Overall, the prevalence of GDM increased in all age groups. Specifically, in total, the standardized annual prevalence increased from 6.02% in 2016 to 7.94% in 2018, with an average annual increase of 5.48% (95% CI 4.67–6.30%). In urban areas, the prevalence increased from 2.82 to 5.07%, while in rural areas, the prevalence increased from 3.29 to 5.46%. The average annual percentage change was greater in rural areas (11.28%, 95% CI 10.07–12.51%) than in urban areas (0.00%, 95% CI − 1.08 to 1.10%).

**Table 4:** Annual prevalence of gestational diabetes mellitus, 2016–2018

Characteristic	2016	2017	2018
Overall			
Mean age at diagnosis (SD), year	30.89 (4.74)	31.59 (4.94)	31.61 (4.85)
Number of cases	30,821	33,399	32,843
Number of births	330,089	323,845	315,318
Prevalence (%)	9.34	10.31	10.42
Standardized prevalence^a^ (%)	6.02	7.98	7.94
Urban areas			
Number of cases	16,427	16,267	15,831
Number of births	109,054	107,029	105,110
Prevalence (%)	15.06	15.20	15.06
Standardized prevalence^a^ (%)	8.24	9.66	9.50
Rural areas			
Number of cases	14,394	17,132	17,012
Number of births	212,430	208,406	201,770
Prevalence (%)	6.78	8.22	8.43
Standardized prevalence^a^ (%)	4.81	7.01	6.94
Urban areas (%)			
20–24 years	5.16	4.71	3.90
25–29 years	12.70	11.43	11.26
30–34 years	21.09	19.91	20.58
35–39 years	30.57	36.04	32.30
40–44 years	18.16	29.36	29.79
45–50 years	2.82	2.87	5.07
Rural areas (%)			
20–24 years	1.90	1.94	2.00
25–29 years	6.08	6.41	6.53
30–34 years	11.17	13.49	14.26
35–39 years	16.21	22.80	21.76
40–44 years	13.70	26.35	24.74
45–50 years	3.29	4.11	5.46

^a^Age-standardized to the 6th population census in Zhejiang province, 2010

**Table 5.** Average annual percentage change of gestational diabetes mellitus prevalence by demographic factors

Characteristic	Average annual percentage change (%)	95% CIs	
		Lower (%)	Upper (%)
Overall			
20–24 years	− 4.38	− 7.27	− 1.40
25–29 years	− 1.35	− 2.66	0.00
30–34 years	5.23	3.83	6.66
35–39 years	8.51	6.70	10.34
40–44 years	28.08	23.92	32.37
45–50 years	32.35	15.08	52.22
20–50 years	5.48	4.67	6.30
Urban areas			
20–24 years	− 12.82	− 16.82	− 8.63
25–29 years	− 6.01	− 7.76	− 4.22
30–34 years	− 1.20	− 3.02	0.65
35–39 years	2.51	0.10	4.97
40–44 years	25.78	19.79	32.06
45–50 years	37.49	10.06	71.74
20–50 years	0.00	− 1.08	1.10
Rural areas			
20–24 years	2.53	− 1.56	6.79
25–29 years	3.60	1.66	5.58
30–34 years	12.70	10.53	14.91
35–39 years	14.79	12.11	17.54
40–44 years	30.04	24.34	36.00
45–50 years	29.07	7.81	54.52
20–50 years	11.28	10.07	12.51

Multivariable Poisson regression models with the calendar year as a continuous variable and adjusting for the other covariates

*CI* confidence interval

## Discussion

In the present study, with data from the population-based diabetes registry system in Zhejiang province of China, we have reported the prevalence and time trends of GDM in women of childbearing age during 2016–2018 and examined the effects of age and residence area on these estimates. Overall, our results suggested that there was a significant increase in the prevalence of GDM, which was also observed by age and residence area. To our knowledge, this is one of the few studies to investigate the prevalence and time trend of GDM in Chinese female population.

During 2016–2018, we showed that the age-standardized overall prevalence of GDM was 7.30% (9.13% in urban areas and 6.24% in rural areas) among Chinese women aged 20–50 years. Although with different GDM diagnostic criteria, study designs, population characteristics, study periods and statistical methods, our results were comparable to those findings from the published studies at present. Using the 1999 WHO criteria, two population-based studies in Tianjin of China reported that the adjusted prevalence of GDM among women living in urban districts between time-points 1999–2008 and 2010–2012 was 4.9% and 8.1%, respectively [18, 19]. In a claims-based analysis conducted in the neighboring country of Korea, the age-adjusted prevalence of GDM in 2009–2011 was 7.5% [14], which was similar to our estimate. In Denmark, a national register-based cohort study of all pregnancies resulting in a birth reported the combined prevalence of GDM was 2.7% (2.4% among Danish born women and 4.3% among immigrant women) between 2004 and 2015 [24]. In contrast, regional data from Aarhus city of Denmark demonstrated that the GDM prevalence was 4.3% during the period of 2013–2016 [25]. Besides, within a population-based cohort study in northeastern Pomerania of Germany, the authors found the rate was 5.1% between 2002 and 2008 [26]. In the last decades, studies on GDM prevalence were also attempted in the United States. For example, using a medical insurance claims-based database within the time frame of 2004–2011, the GDM prevalence was found to be 6.29% among 839,792 pregnancies [27]. More recently, in the National Health and Nutrition Examination Surveys 2007–2014, the prevalence of GDM among U.S. women aged ≥ 20 years was found to be 7.6% [28].

In our study, the age-standardized annual prevalence of GDM increased from 6.02% in 2016 to 7.94% in 2018, with the average annual percentage change of 5.48%, which was similar to other results in previous literature. Of 156,144 women who gave birth in southern Sweden, a study showed that the prevalence of GDM was 1.9% in 2003 and 2.6% in 2012, which rose at an annual rate of 3.4% [29]. Another study conducted in Sydney of Australia also indicated that the average prevalence of women with GDM increased from 8.7% in 2011 to 13.9% in 2017, an increase of 9% annually [30]. Although reasons for the rising trend of GDM in our study were unclear, there were several possible explanations with respect to the increasing prevalence of the undiagnosed diabetes, GDM related factors, and a declining missing report rate of the diabetes surveillance system. Nationally representative surveys have indicated that the prevalence of undiagnosed diabetes in Chinese women rose from 5.2% in 2007 to 6.1% in 2013 [31, 32]. When these diabetes patients were firstly detected during pregnancy, they were usually diagnosed as GDM cases. According to convincing evidence, the higher pre-pregnancy body mass index (BMI) was one of the strongest risk factors of GDM and the increasing prevalence of GDM might reflect the current patterns of increasing obesity [33, 34]. Four national surveys showed that the prevalence of obesity among Chinese women aged 20–59 years increased from 7.5% in 2000, to 8.4% in 2005, 9.2% in 2010, and 9.3% in 2014, with the annual percentage change of 0.13% [35]. Physical activity was found to be associated with odds of GDM [36, 37], while Chinese national surveys showed that the percentage of women meeting the minimum leisure-time physical activity increased over time (15.9% in 2002, 16.9% in 2005, and 22.1% in 2014) [35]. GDM was also reported to be associated with higher parity [12]. However, according to the China Kadoorie Biobank (CKB) study conducted during 2004–2008, the mean parity fell (urban: 4.9–1.1; rural 5.9–1.4) among 300,000 Chinese women aged 30–79 years [38]. Besides, advanced maternal age is an independent risk factor for GDM [39, 40] and based on our analysis, the mean age at GDM diagnosis increased from 30.89 to 31.61 years during 2016–2018. Notably, in 2015, the Chinese government announced that all couples would be allowed to have two children. Based on a national cross-sectional study in 2016–2017, four in ten women of childbearing age planned to have a second child, especially for women of an advanced age [41]. Except for the contributions of the diabetes related factors, the rising trend of GDM might also be attributed to the efficiency change of the diabetes surveillance system. In 2009, we begun to use the completely computerized diabetes surveillance system, with more convenient case-reporting procedure and stricter quality control measures, the rate of missing report was diminishing.

Age disparities in GDM prevalence have been observed in previous reports [14, 42]. In accordance with these studies, our results showed higher prevalence of GDM in women with advanced age. Besides, we found that the prevalence of GDM was slightly higher in urban area than that in rural area, consistent with the findings in an India study [43], but different from another national study in Turkey showing that there was no difference in GDM prevalence between urban and rural regions [44]. Furthermore, importantly, our analysis showed a greater average annual increase in prevalence in rural area than in urban area, which suggested a more rapid growth of GDM in rural area and necessary intervention measures must be implemented.

Our study had several strengths. This is one of the few studies to investigate the prevalence and time trend of GDM among Chinese female population in mainland China. Data analyzed in the study were extracted from the computerized diabetes surveillance system, which was continually able to monitor the odds of GDM among province-wide population. With a relatively larger sample of 97,063 cases diagnosed by certificated health practitioners, the prevalence and time trends of GDM during 2016–2018 were reported eventually. Meanwhile, some limitations must be considered in this study. The greatest weakness of the study is that our reported data on GDM prevalence might be under-estimated. First, due to the lack of direct data on births, we used the number of children to evaluate the number of births, with neglecting the small probability of a multiple birth. Second, we have conducted several rounds of under-reporting investigations and based on the relevant data in 2007 and 2016 [21, 45], the under-reporting proportion of diabetes (including GDM) was reported to be 45.07% and 22.05%, which indicated that our reported rate was underestimated. Third, there are different GDM diagnostic criteria at present and may influence the GDM prevalence across the studies. In multivariable Poisson regression models, we adjusted for age, residence area and calendar year as possible as we can, while other GDM related factors including obesity status, physical activity level and dietary behaviors were not considered. Besides, the number of GDM cases in some specific years was relatively small, which would decrease the statistical power to explore the time trend of GDM in the analysis.

## Conclusions

In conclusion, this study suggested a significant increase in the prevalence of GDM among Chinese female population in Zhejiang province during 2016–2018, especially in women characterized by advanced age and rural areas.

## Data Availability

The datasets generated and/or analyzed during the current study are not publicly available due individual privacy information protection but are available from the corresponding author on reasonable request.

## Abbreviations

GDM: Gestational diabetes mellitus
IDF: International Diabetes Federation
CDC: Disease control and prevention
PRR: Prevalence rate ratio
CI: Confidence interval
BMI: Body mass index
CKB: China Kadoorie Biobank
